# Care coordination and patient safety outcome: a graph-based approach

**DOI:** 10.1038/s44401-025-00020-9

**Published:** 2025-05-08

**Authors:** Hongyu Chen, Yu Huang, Changyu Yin, Xing He, Ronald L. Ison, Liliana L. Bell, Raymond A. Opoku, Zhe Jiang, Serena Jingchuan Guo, Mei Liu, Patrick J. Tighe, Jiang Bian

**Affiliations:** 1 Department of Health Outcomes and Biomedical Informatics, University of Florida, Gainesville, FL USA; 2 Department of Biostatistics and Health Data Science, Indiana University, School of Medicine, Indianapolis, IN USA; 3 Regenstrief Institute, Indianapolis, IN USA; 4 Department of Anesthesiology, College of Medicine, University of Florida, Gainesville, FL USA; 5 Quality and Patient Safety Initiative, College of Medicine, University of Florida, Gainesville, FL USA; 6 Department of Computer & Information Science & Engineering, University of Florida, Gainesville, FL USA; 7 Department of Pharmaceutical Outcomes and Policy, College of Pharmacy, University of Florida, Gainesville, FL USA; 8 Indiana University Health, Indianapolis, IN USA

**Keywords:** Information technology, Outcomes research

## Abstract

Predicting postoperative adverse events and managing the associated risk factors is crucial for patient safety. Care coordination, also known as provider team interactions, significantly impacts outcomes, yet few studies have explored this link and applied it to risk prediction. To address this, Medical Heterogeneous Graphs for Patient Safety analysis (MedHG-PS), a novel graph-based framework that simultaneously models complex relationships among patient characteristics, provider interactions, and patient transfer records was proposed in this study. Evaluated on a real-world dataset with 102,768 patients from the University of Florida Health Integrated Data Repository, MedHG-PS outperforms state-of-the-art methods, achieving an AUC above 0.90 and up to a 20% improvement in recall for three major postoperative outcomes—prolonged length of stay (PLOS), 30-day mortality, and 90-day mortality. By using meta-path analysis (MPA), SHapley Additive exPlanations (SHAP) and Local Interpretable Model-agnostic Explanations (LIME), MedHG-PS identifies key predictive features, such as patient transfers highly influencing PLOS, whereas provider interactions affect mortality risks. This study highlights how care coordination can be modeled at scale using EHRs and can affect patient care safety outcomes—an important aspect of an automated and rapid-learning health system.

## Introduction

Patient safety refers to the absence of preventable harm to patients and the minimization of the potential risks associated with healthcare procedures^1^. Despite the concerted efforts and investments made by federal and local governments, private agencies, and concerned institutions to improve patient safety, studies persistently reveal unfavorable patient safety outcomes^2,3^. The emergence of real-world data (RWD), including electronic health records (EHRs), and developments of artificial intelligence (AI)/machine learning (ML) techniques offer a valuable opportunity to develop personalized prediction models that can assess patient safety outcomes^4–6^. In the perioperative setting, a potential strategy for improving care quality is to build AI/ML models to predict the risk of postoperative adverse events^7^ (e.g., mortality) and manage the associated risk factors. This proactive approach not only promotes patient safety but also improves the workplace environment of healthcare delivery organizations^8^.

Most existing models primarily rely on demographic variables (e.g., age, sex, and race) and clinical factors (e.g., anthropometric variables, physical and laboratory examinations, questionnaires, and lifestyles)^9^. Various outcome measures, such as mortality, postoperative length-of-stay^10,11^, and readmission, are often considered as the outcomes of interest for assessing care quality and patient safety. These measures reflect the impact of healthcare services or interventions on patient health directly or indirectly^12^ Yet, such outcomes are influenced by numerous factors, many of which are beyond the control of providers. This variability across patients makes accurate prediction a significant challenge.

In real-world settings, the communication and interactions among a patient’s healthcare team, also known as care coordination, have a significant impact on the quality of care and patient safety outcomes, particularly in perioperative settings^13^. Despite its importance, only a limited number of studies^14,15^ (see detailed literature review in Supplementary Section 1) have explored how such interactions affect patient safety outcomes. Incorporating care team coordination—which can be modeled as care coordination networks—into outcome prediction models could enhance predictive accuracy and provide explainable insights to help the care team improve the quality of care.

In this study, a graph-based analytic framework, called medical heterogeneous graphs for patient safety analysis (MedHG-PS), was proposed. This framework incorporates heterogeneous graph neural networks (HGNN) to simultaneously model patient demographics characteristics, along with perioperative information, transfer records, and provider team information during the stay, to predict patient postoperative safety outcomes. An interpretable and efficient heterogeneous graph convolutional network (ie-HGCN)^16^ was used as the backbone model of the MedHG-PS. This model effectively analyzes heterogeneous graphs, identifying the most relevant meta-paths—patterns representing semantic relationships between nodes in the graph (e.g., patients and providers)—for specific tasks. Its ability to enhance accuracy and interpretability^16^ makes it suited for healthcare application^17^. Furthermore, by integrating explainable AI (XAI) techniques into the MedHG-PS, key factors among clinical factors to care coordination that contribute most to the predictions were identified.

A series of experiments were conducted on a cohort (*n* = 102,768) to validate the proposed framework, using EHRs from the University of Florida (UF) Health Integrated Data Repository (IDR). The framework was comprehensively evaluated against various baseline models, including ML techniques combined with network science approaches, for multiple outcome predictions. The key contributions of this study are as follows: (1) the development of a graph-based analytic framework, MedHG-PS, for modeling care coordination using real-world data; (2) the evaluation of MedHG-PS in predicting patient safety outcomes; (3) the use of XAI techniques to identify key factors influencing patients’ outcomes—an important aspect of an automated and rapid-learning health system.

## Results

### Descriptive statistics of the study cohort

The cohort included 136,647 surgical encounters, with 102,768 adult patients (Table 1). The mean age was 55.72 (±18.01) years, and 60.37% were women. Of the cohort, 64.73% were Non-Hispanic White (NHW), 12.81% were Non-Hispanic Black (NHB), 4.64% were Hispanic, and 17.82% were other races/ethnicities. For three outcomes, based on encounter-level statistics, 25.00% present prolonged length of postoperative hospitalization (i.e., greater than 75th percentile, 6 days), 3.16% and 5.32% encounters have 30-day and 90-day mortality records. Statistically significant differences in patient outcomes were identified across age, sex, and race-ethnicity groups (*p* value < 0.01). The detailed statistics of the study cohort are shown in Supplementary Table S2. The missing data rates for non-binary features in this study are summarized in Supplementary Table S3. Consistent with previous research, this dataset generally exhibits low missing data rates, with most variables missing less than 2%. However, higher missing rates for certain variables were observed: the Charlson Comorbidity Index (CCI) had the highest missing rate at 28.49%, followed by body mass index (BMI) at 9.52%, and height at 9.22%.

**Table 1:** Cohort information

		Patients (Percentage)
Patient-level	Total	102,768
	Age	55.72 (±18.01)
	Sex	
	Female	62,042 (60.37%)
	Male	40,726 (39.63%)
	Race-Ethnicity	
	NHW	66,525 (64.73%)
	NHB	13,161 (12.81%)
	Hispanic	4765 (4.64%)
	Other	18,317 (17.82%)
Encounter-level	Total	136,647
	PLOS	
	Yes	34,156 (25.00%)
	No	102,491 (75.00%)
	30-day mortality	
	Yes	4319 (3.16%)
	No	132,328 (96.84%)
	90-day mortality	
	Yes	7274 (5.32%)
	No	129,373 (94.68%)

### Prediction model of patient safety outcome

**Fig. 1:**
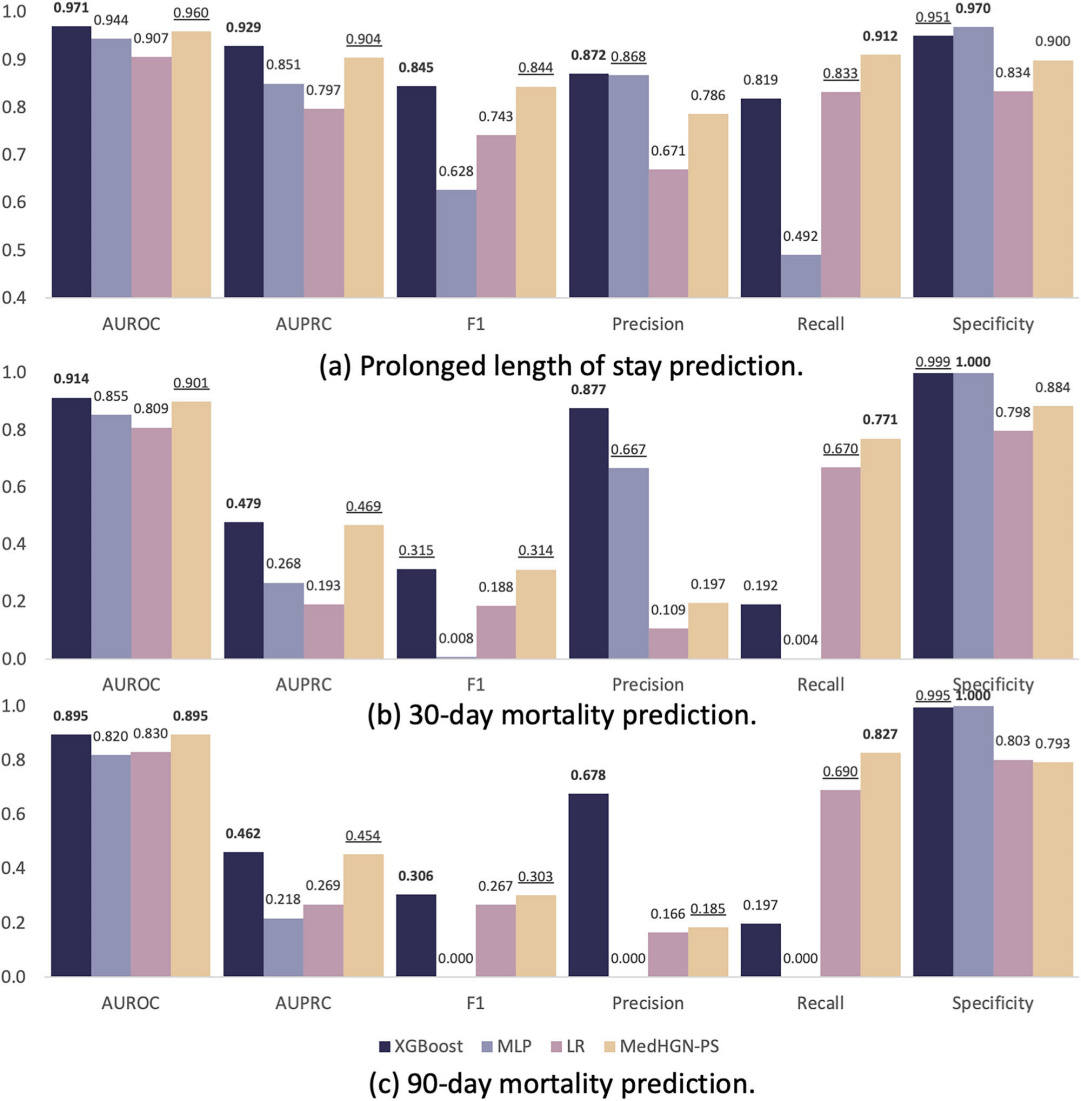
Model performance assessment of MedHG-PS and baseline models. The AUROC, AUPRC, F1-score, precision, recall, and specificity of MedHG-PS, XGBoost, MLP, and LR in predicting **a** PLOS, **b** 30-day mortality, and **c** 90-day mortality.

Figure 1 shows the prediction performance of three tasks among four models. As shown in Fig. 1a, MedHG-PS shows an area under the receiver operating characteristic (AUROC) of 0.960 (95% confidence intervals [95% CI]: 0.956–0.963), an area under the precision-recall curve (AUPRC) of 0.904 (95% CI: 0.895–0.913), an F1-score of 0.844, a precision of 0.786, a recall of 0.912, and a specificity of 0.900. Compared to the baseline models, particularly the best-performing model, Extreme Gradient Boosting (XGBoost), MedHG-PS demonstrates higher recall performance while maintaining comparable metrics for other measures. Across these three tasks, the outcome distributions varied. While 30-day and 90-day mortality predictions are more imbalanced compared to PLOS predictions, MedHG-PS excels in achieving high recall rates for handling different tasks. Figure 1b visualizes the performance in predicting 30-day mortality, where MedHG-PS achieved the highest recall (0.771), outperforming the best traditional ML models by over 15%. For the 90-day mortality prediction task (Fig. 1c), MedHG-PS further demonstrates its strengths, outperforming the ML models by at least 20% in recall rates. These results confirm the robustness of MedHG-PS across both balanced and imbalanced prediction tasks, making it particularly suitable for predicting negative events with potentially irreversible consequences^18^.

The impacts of different features on model performance across these three tasks were also investigated, as detailed in Supplementary Tables S4–S6. For the PLOS prediction task, using only demographic and perioperative information yielded satisfactory results with XGBoost, achieving an AUROC of 0.964 (95% CI 0.961–0.967), AUPRC of 0.905, F1-score of 0.827, precision of 0.843, recall of 0.812, and specificity of 0.938. Although the differences in AUROC is not significant compared with demographic and perioperative information only mode (*p* value = 0.06), incorporating provider interaction and patient transfer information improved the recall to 0.912 when using MedHG-PS. Notably, even without patient-specific demographic or perioperative information, the provider team alone demonstrated predictive capabilities, with an AUROC of 0.694, a recall of 0.640, and an F1-score of 0.503—significantly outperforming XGBoost (AUROC = 0.645; recall = 0.178; F1-score = 0.272).

For the more challenging tasks of 30-day and 90-day mortality predictions, relying solely on demographic and perioperative information resulted in suboptimal recall performance of 0.592 and 0.674, respectively. MedHG-PS significantly improved the recall rates to 0.771 and 0.827 without compromising AUROCs (*p* value = 0.12 and 0.70), AUPRCs, and F1-scores performance. The importance of provider teams with MedHG-PS was even greater for these mortality prediction tasks (Supplementary Tables S5 and S6), achieving AUROCs of 0.721 and 0.701, recalls of 0.682 and 0.718, and F1-scores of 0.124 and 0.155, compared with AUROCs of 0.649 and 0.603, recalls of 0.000 and 0.000, and F1-scores of 0.000 and 0.000 for XGBoost, respectively. In summary, provider interaction and patient transfer information play a crucial role in enhancing the prediction of mortality outcomes, particularly for more complex and imbalanced tasks.

A series of ablation studies were performed to examine the models’ robustness to missing data and class imbalance. In the missingness experiment, key variables—BMI, height, CCI, and ASA-PS—were manually masked. This led to modest declines in predictive performance for both PLOS and 30-day mortality outcomes. For PLOS prediction, performance metrics decreased as follows: AUROC by 0.009, AUPRC by 0.048, F1-score by 0.002, precision by 0.018, recall by 0.020, and specificity by 0.014. For 30-day mortality prediction, a slight increase in AUROC by 0.02, accompanied by decreases in AUPRC (0.008), F1-score (0.025), precision (0.021), recall (0.029), and specificity (0.022) were observed. The impact was more significant for 90-day mortality prediction, with performance metrics showing declines ranging from 0.053 to 0.255. Contrary to expectations, resampling techniques failed to enhance model performance, even in highly imbalanced scenarios. Instead, all models experienced decreased overall performance. For PLOS, undersampling slightly reduced all metrics, while oversampling significantly impacted the AUPRC, decreasing it by 7.9%, with other metrics declining between 0.2% and 6.6%. For 30-day mortality, undersampling significantly affected performance, with metrics decreasing by 2.1% to 66.1%, while oversampling improved the F1-score to 0.361 but significantly sacrificed recall (0.274), reducing the practical utility of the MedHG-PS. Similarly, for 90-day mortality, undersampling showed moderate performance impacts, while oversampling improved precision at the expense of recall. On the other hand, although less prominent, ML models also experienced similar negative impacts with resampling. Detailed statistics can also be found in Supplementary Tables S4–S6.

To examine the computational efficiency of MedHG-PS, Multiply-Accumulate Operations (MACs) and inference time were calculated. With only 3.2 million parameters, MedHG-PS demonstrated low computational cost, requiring only 75.85 million MACs. When performing inference on a single A100 GPU, MedHG-PS required only 0.03 s to generate predictions for an individual patient.

### Explainable AI to identify important feature contributing to predicting patient safety outcome

**Fig. 2:**
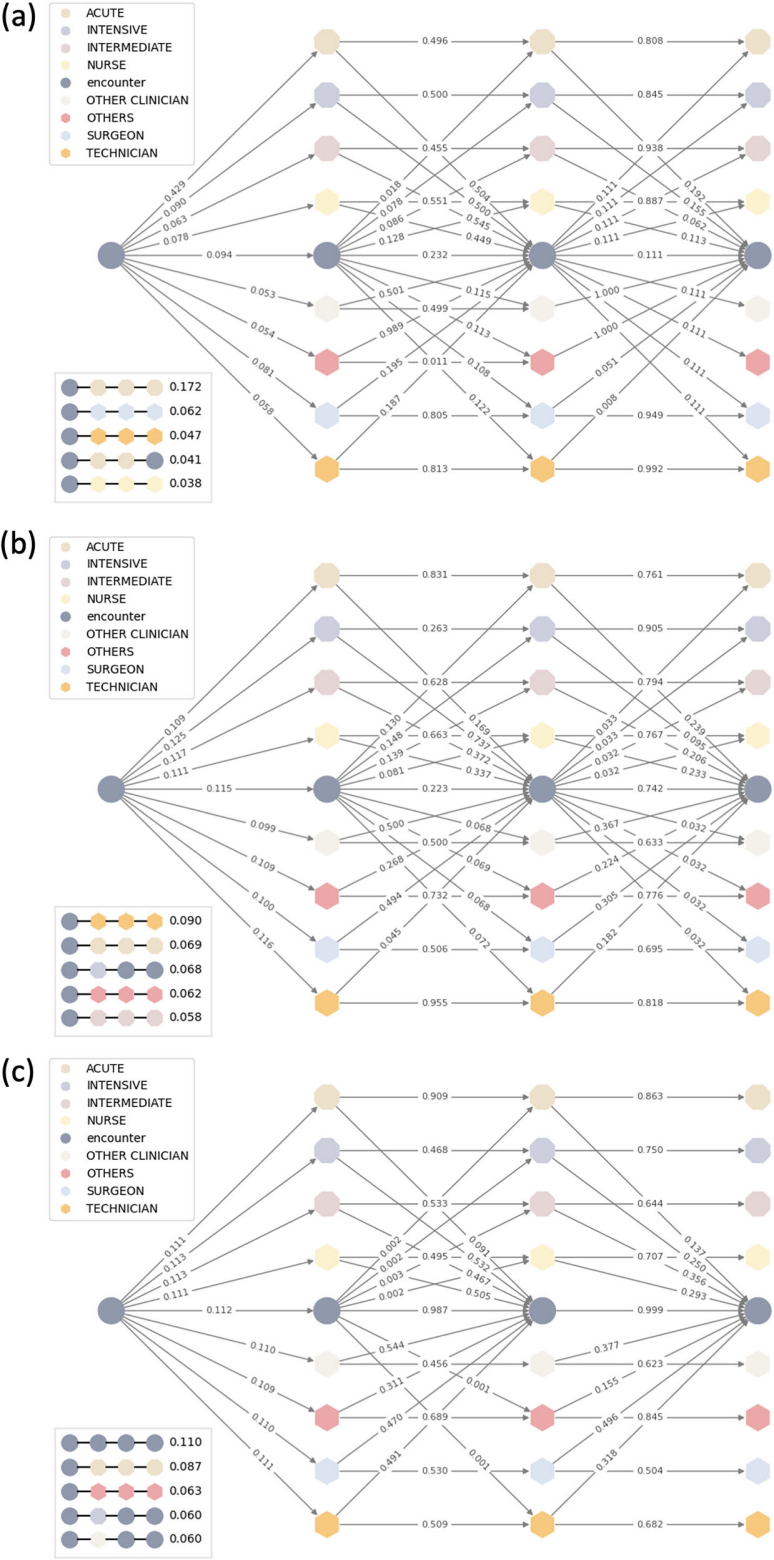
Meta-path importance analysis. The plot visualized the contribution of each meta-path to the MedHG-PS when predicting **a** PLOS, **b** 30-day mortality, and **c** 90-day mortality.

Figure 2 shows the meta-paths generated by MedHG-PS using a three-layer ie-HGCN, comparing the importance of encounter factors (i.e., demographics and perioperative information), provider-provider interactions, and patient transfer information. For the PLOS prediction (Fig. 2a), the top five meta-paths were identified: Encounter-Acute Care Unit, Encounter-Surgeon, Encounter-Technician, Encounter-Acute Care Unit-Encounter, and Encounter-Nurse. The most influential factor was the level of care, with an importance score of 0.172—significantly higher than the second-ranked meta-path. Notably, two of the top five meta-paths involved the Acute Care Unit, underscoring its critical role in predicting PLOS. Additionally, interactions with surgeons and technicians, such as perfusionists, also contributed significantly to PLOS predictions. The results of 30-day mortality prediction task (Fig. 2b) suggest that provider teams and operational risks are key factors in predicting 30-day mortality. The complexity of operations and the skill levels of providers, as represented by the Technician and Surgeon nodes, play a critical role. In contrast, for the 90-day mortality prediction task, the direct contributions of providers involved in operations diminished. Instead, nodes associated with perioperative teams, including Other Clinicians and others (e.g., anesthesiologists and observers), played a more prominent role, emphasizing their importance in predicting longer-term outcomes.

**Fig. 3:**
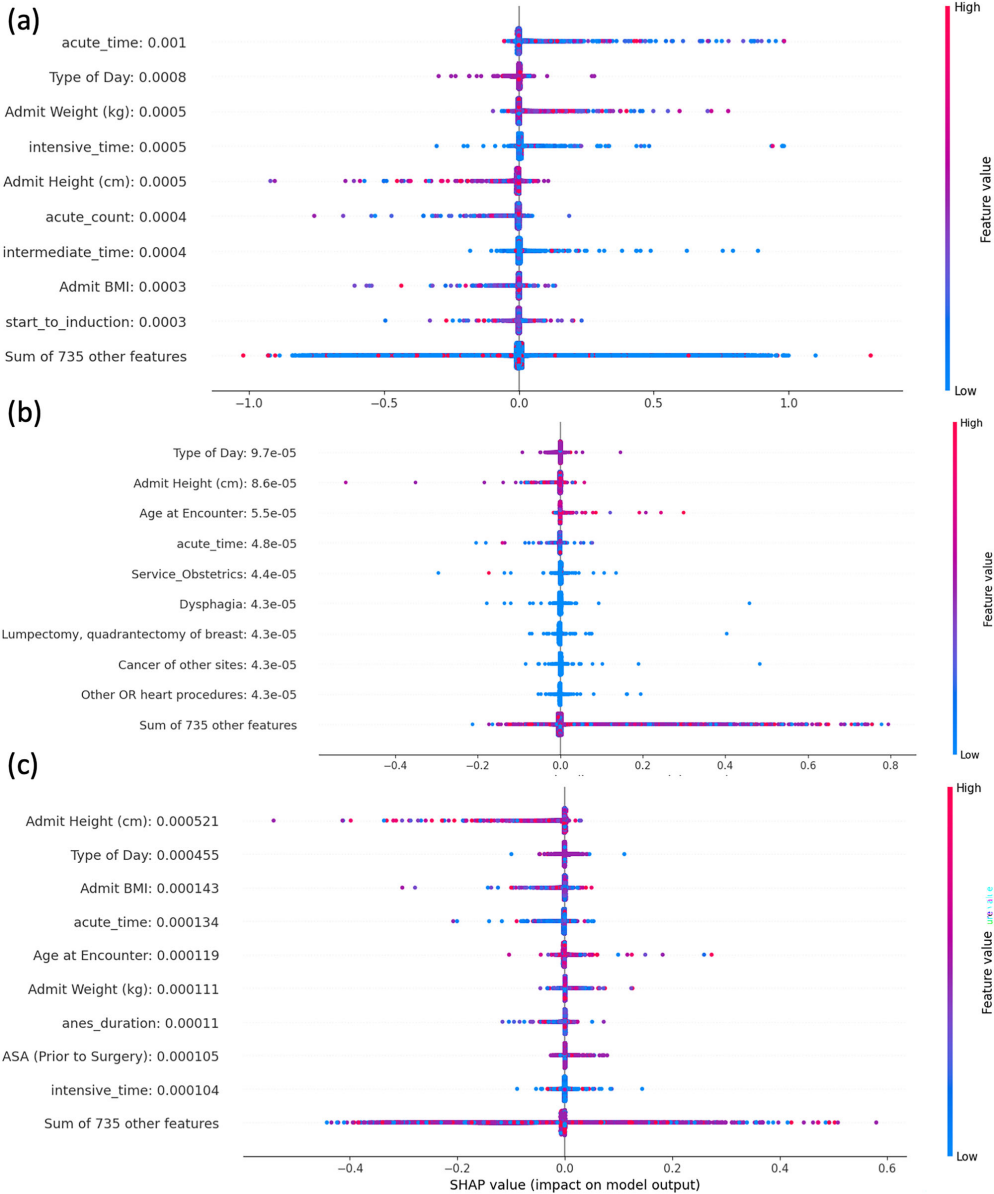
Feature importance analysis with SHAP values. The SHAP plot visualized the contribution of each feature to the MedHG-PS when predicting **a** PLOS, **b** 30-day mortality, and **c** 90-day mortality.

SHapley Additive exPlanations (SHAP) analysis was applied to examine the contributions of encounter information, including demographics and perioperative features, to the model’s predictions. For the PLOS prediction task (Fig. 3a), the most significant feature was time spent in the acute care unit, which positively influenced predictions (longer stays correlated with PLOS). Other important features included type of day (weekday or weekend), admission weight, and BMI. In the 30-day (Fig. 3b) and 90-day mortality prediction tasks (Fig. 3c), similar features were identified. However, SHAP values for these features were comparable, indicating they contributed equally without any dominant features. These findings align with the meta-path analysis (MPA), confirming that for mortality predictions, provider coordination and patient transfer records add substantial predictive value to the models.

**Fig. 4:**
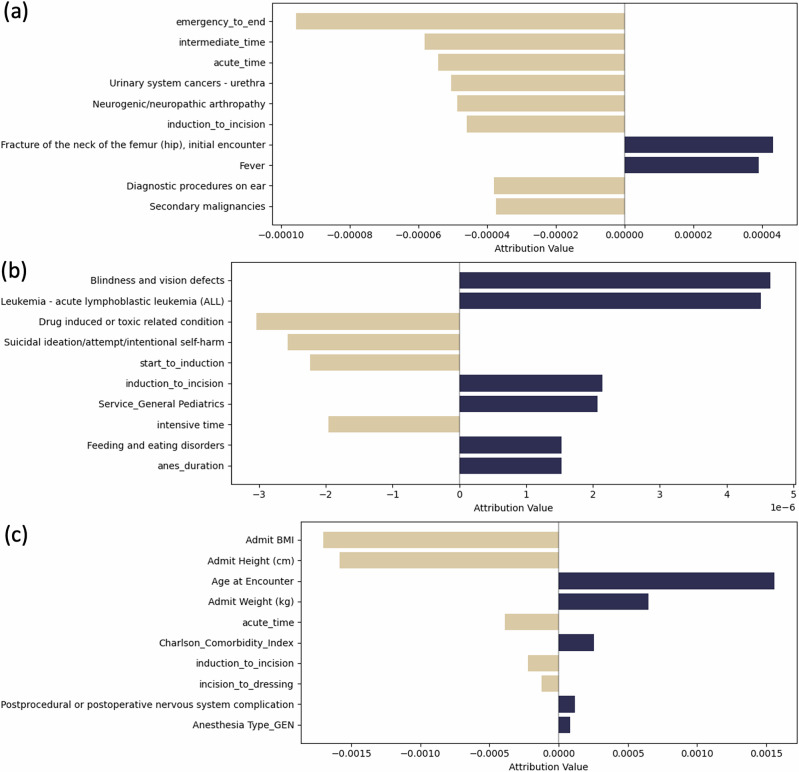
Feature importance analysis with LIME values. The LIME plot visualized the contribution of each feature to the MedHG-PS when predicting **a** PLOS, **b** 30-day mortality, and **c** 90-day mortality.

Local Interpretable Model-agnostic Explanations (LIME) analysis complemented the SHAP findings by providing further insights into feature contributions across prediction tasks. For PLOS prediction (Fig. 4a), hip fracture and fever emerged as significant positive contributors, while several time-related variables (emergency to end, induction to incision), care unit durations (intermediate time, acute time), urinary system cancers, diagnostic ear procedures, and secondary malignancies were associated with reduced PLOS risk. In the 30-day mortality model (Fig. 4b), blindness/vision defects and acute lymphoblastic leukemia (ALL) demonstrated the strongest positive associations with mortality outcomes. Additional mortality risk factors included surgical timing variables (induction to incision), service type (General Pediatrics), feeding disorders, and anesthesia duration. Conversely, drug-induced conditions, suicidal ideation/self-harm, start to induction timing, and intensive care duration appeared to reduce 30-day mortality risk, potentially reflecting increased monitoring and intervention for these conditions. The 90-day mortality prediction model (Fig. 4c) reinforced the SHAP analysis findings, with age at encounter serving as the predominant risk factor, followed by weight, CCI, and postoperative neurological complications. Protective factors included BMI, height, acute care duration, and surgical timing metrics (induction to incision, incision to dressing). The consistency between LIME and SHAP attribution values across features, particularly for 30-day mortality prediction, strengthens confidence in the feature importance assessments and suggests robust model interpretability.

## Discussion

In this study, MedHG-PS, a heterogeneous graph-based framework to model care coordination and predict postoperative safety outcomes, was developed. MedHG-PS learns latent relationships and embeddings from perioperative information, provider team details, and patient transfer records using heterogeneous graphs derived from EHR data. It supports multiple tasks, including PLOS prediction, 30-day, and 90-day mortality predictions. The framework was evaluated on a cohort of 102,768 real-world surgical patients from the UF IDR EHR dataset. Compared to traditional ML models combined with network science techniques, MedHG-PS demonstrated up to a 20% improvement in recall rates across the three prediction tasks. Given the potentially devastating consequences of negative outcomes, recall is the most critical metric, particularly within learning health systems. This is especially important for smaller hospitals, which may experience higher mortality^19^

The findings of this study have implications for clinical practice, particularly in the context of developing automated, real-time decision support tools. Length of stay (LOS) is an important factor to evaluate the efficiency of a healthcare system. PLOS could increase costs for both patients and healthcare systems while potentially reducing quality of care and patient satisfaction^20^. The 30-day and 90-day mortality are widely used patient safety outcome measures employed to assess and compare healthcare quality for benchmarking and pay-for-performance initiatives^21,22^. By incorporating care coordination data into outcome prediction models, healthcare organizations can more accurately identify patients at risk of adverse events, enabling more targeted and timely interventions^23,24^. For example, accurate PLOS prediction has the potential to guide bed management, discharge planning, and resource allocation, ultimately improving the efficiency of hospital operations and patient throughput^25^. Similarly, the high recall achieved by MedHG-PS in predicting 30-day and 90-day mortality highlights its potential utility in identifying high-risk patients who may benefit from early interventions or closer postoperative monitoring. This capability is especially critical in surgical settings, where timely actions can significantly reduce morbidity and mortality.

The MedHG-PS model provides comprehensive analysis using Meta-Path Analysis (MPA), SHAP, and LIME for different purposes. MPA investigates the importance of encounter information (e.g., demographic and perioperative features), care coordination (e.g., provider-provider interactions), and patient transfer records. Meanwhile, SHAP and LIME highlights the most important factors within the encounters themselves. Based on MPA, several findings were obtained for three outcomes: (1) For PLOS prediction, patient transfer records were identified as the most important factors; (2) For 30-day mortality prediction, care coordination between technicians and surgeons played a vital role; and (3) For 90-day mortality prediction, perioperative teams (e.g., anesthesiologists and observers) emerged as the primary contributors. In PLOS prediction, acute care units were the most influential feature based on both attention coefficients and SHAP analysis. This aligns with clinical intuition, as preoperative stays in acute care units typically indicate more severe or complex conditions, leading to extended recovery times and increased resource utilization^26^. SHAP analysis also revealed that the type of day (commonly referred to as the “weekend effect”) was the second most influential feature for PLOS prediction. This effect has been well-documented in healthcare literature, showing that patients admitted or undergoing surgery on weekends tend to have longer hospital stays^27^. Additionally, BMI and admission weight were significant factors, consistent with prior studies that associate higher BMI with longer PLOS^28,29^. For mortality prediction, several features stood out. The weekend effect was a key factor, which is consistent with findings from prior studies that have demonstrated higher mortality rates for patients admitted or treated on weekends^30^. Another prominent feature in the mortality models was admission age, which had a strong positive influence on both 30-day and 90-day mortality predictions. Age has long been recognized as a critical determinant of surgical outcomes, with older patients being more vulnerable to postoperative complications and mortality^31,32^.

The LIME analysis conducted in this study provides complementary insights to MPA and SHAP findings, further illuminating the feature-level contributions to patient safety outcomes. For PLOS prediction, LIME suggested that specific clinical conditions like hip fractures and fever significantly increase likelihood of extended hospitalization. This aligns with established clinical knowledge that hip fractures often require complex surgical interventions and rehabilitation protocols, typically necessitating extended inpatient care^33^. In the 30-day mortality prediction model, LIME analysis revealed ALL emerged as one of the strongest mortality predictors. ALL’s strong association with 30-day mortality aligns with the known high-risk nature of this malignancy^34^. Counterintuitively, LIME identified suicidal ideation and drug-induced conditions as protective factors against 30-day mortality. This paradoxical finding likely reflects the intensive monitoring and specialized care these patients receive rather than a direct biological protective effect, highlighting how healthcare delivery patterns can influence predicted outcomes. For 90-day mortality, LIME analysis reinforced the primacy of age as the dominant risk factor, consistent with the SHAP findings. The CCI’s strong positive influence further validates the model’s alignment with clinical expectations, as this index was specifically designed to predict mortality^35^. The negative association between BMI and 90-day mortality aligns with the “obesity paradox” observed in some surgical populations, where moderate obesity appears protective against longer-term mortality despite increasing other complications^36^.

However, this study has several limitations. First, the data were sourced from a single healthcare system, which may restrict the generalizability of these findings, as they are influenced by specific geographical factors (i.e., Florida). Second, several features (e.g., Insertion, revision, replacement, removal of cardiac pacemaker or cardioverter/defibrillator, encounter for mental health conditions, sexually transmitted infections) had limited sample sizes, potentially introducing bias into the model. Future research should focus on validating MedHG-PS across diverse healthcare systems to assess its broader applicability. In addition, incorporating advanced feature engineering techniques can help in mitigating potential bias in the model. Specifically, for mortality prediction, ML models demonstrate high precision in mortality prediction but suffer from low recall, indicating they accurately identify positives while failing to capture a substantial portion of at-risk patients. Conversely, the MedHG-PS model achieves high recall, successfully capturing most mortality cases, albeit with a slightly higher rate of false positives. This trade-off suggests that developing ensemble methods that integrate MedHG-PS with traditional ML models could be a promising direction for future research. Such hybrid approaches may leverage the high precision of ML models and the superior recall of MedHG-PS to achieve more balanced and clinically useful predictive performance. Additionally, findings from feature masking and resampling experiments indicate that advanced data augmentation and imputation strategies —particularly those incorporating care coordination information—should be further explored to address the inherent imbalance in outcome distributions.

In conclusion, this study highlights the significant potential of GNN-based approaches in modeling care coordination and predicting postoperative safety outcomes. This method has proven to be a promising tool for predicting multiple patient safety outcomes with high recall rates. Leveraging EHR data, the MedHG-PS holds great potential for integration into EHR systems and clinical workflows.

## Methods

### Data and study design

**Fig. 5:**
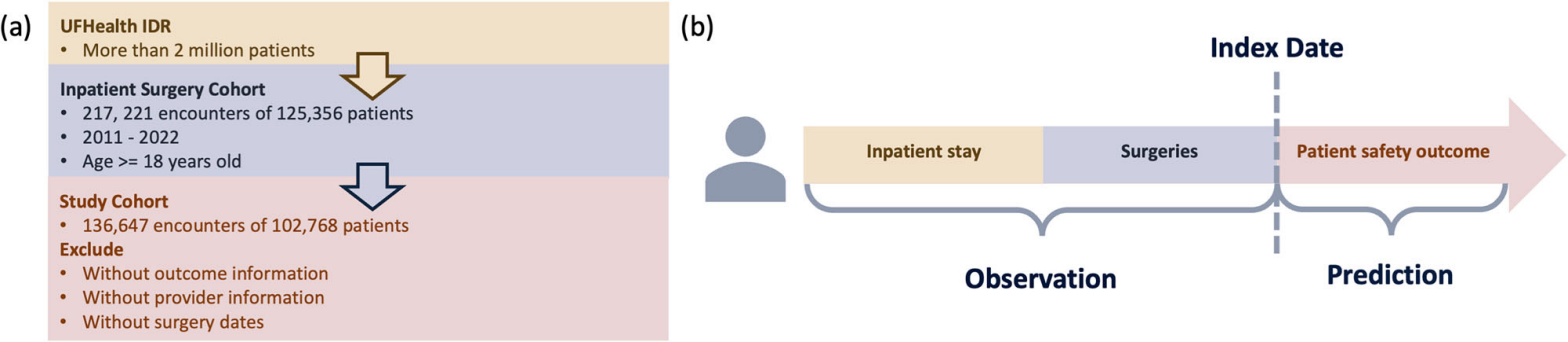
Processing workflow of the UF Health integrated data repository inpatient surgery cohort and the patient timeline. **a** Study cohort construction process. **b** Patient timeline. The study cohort consists of adult inpatient surgery patients with documented provider and outcome information. Risk factors are observed during the inpatient stay, prior to the completion of the last surgery. The model is designed to predict patient safety outcomes based on the pre-surgery observation period.

A retrospective study was conducted using EHR data from UF Health IDR, an enterprise data warehouse integrating different patient information systems across the UF Health system. In this study, 125,356 adult patients with 217,221 inpatient encounters who underwent at least one surgical procedure between 2011 and 2022 were initially identified. Patients were excluded if they lacked (1) outcome information (i.e., LOS or mortality), (2) provider information, or (3) recorded surgery dates. After applying the exclusion criteria, the final cohort comprised 136,647 inpatient encounters of 102,768 patients. For this study, the index date was defined as the date of the last surgical procedure within each hospital encounter. Predictors were extracted for each patient during their inpatient stay to the point of discharge, while outcomes were assessed after the index date. The data extraction process is visualized in Fig. 5. This study was conducted in accordance with the ethical principles outlined in the Declaration of Helsinki. This study was approved as exempt by the University of Florida Institutional Review Board (IRB) under protocol number IRB202202634. The research involved the use of de-identified EHR data to ensure participant privacy and confidentiality.

### Study outcomes

Three study outcomes were validated in this study: PLOS, 30-day and 90-day all-cause mortality after the index date. To determine PLOS, the 75th percentile was used as the cut-off threshold according to the related works^37–41^. The 30-day and 90-day mortalities are two important criteria set by the Centers for Medicare & Medicaid Services (CMS) for hospitals^42^.

### Covariates

Various patient demographic characteristics recorded at the time of admission were incorporated as predictive variables, including age, weight, height, BMI, race, ethnicity, sex, and marital status.

Clinical factors and calendar-related factors during the patient’s perioperative period were extracted. For clinical factors, comorbidities, the CCI^35^, the ASA-PS^43^, surgery services received, procedure interval, anesthesia-related information, and the presence of delirium^44^ were included. The procedure intervals, including the durations between various operative stages: from room start to induction, from induction to incision, from incision to dressing, from dressing to emergency, from emergency to room end, and duration from the anesthesia start to end were also extracted. Anesthesia-related information was incorporated, including type of anesthesia, and occurrence of anesthesia block. Calendar-related factors, which can potentially impact health quality, were also considered; for example, the type of day (weekday or weekend) may interact with provider shifts, influencing healthcare quality^45^. These features included the day of week, type of day, admission source, and care unit type.

Preoperative ICU stay has been recognized as a risk factor for adverse outcomes^46^. Accordingly, records of emergency admissions, preoperative LOS across various care unit types (i.e., intermediate, intensive, and acute), and frequency of transfers between these units were incorporated as predictive features.

A key focus of this study is to discover the impact of care team coordination on outcomes. Therefore, provider information (e.g., expertise type) were included and the interactions among providers within the same encounter and across different encounters were analyzed.

### The proposed framework

The proposed analysis framework, MedHG-PS, has three steps: (1) graph construction, (2) GNN modeling, and (3) feature contribution exploration. The inputs to this framework include demographics, perioperative information, transfer records, and provider team details. Before loading inputs to the framework, the raw data was preprocessed by filtering out outliers, standardizing continuous features, and applying one-hot encoding to categorical variables. Missing data were addressed by assigning “Unknown” to missing categorical variables and imputing missing continuous variables using their mean values. To address the imbalanced nature of patient safety outcomes, resampling strategies were further assessed. For each task, data balancing was achieved by either randomly removing negative samples or oversampling positive samples.

**Fig. 6:**
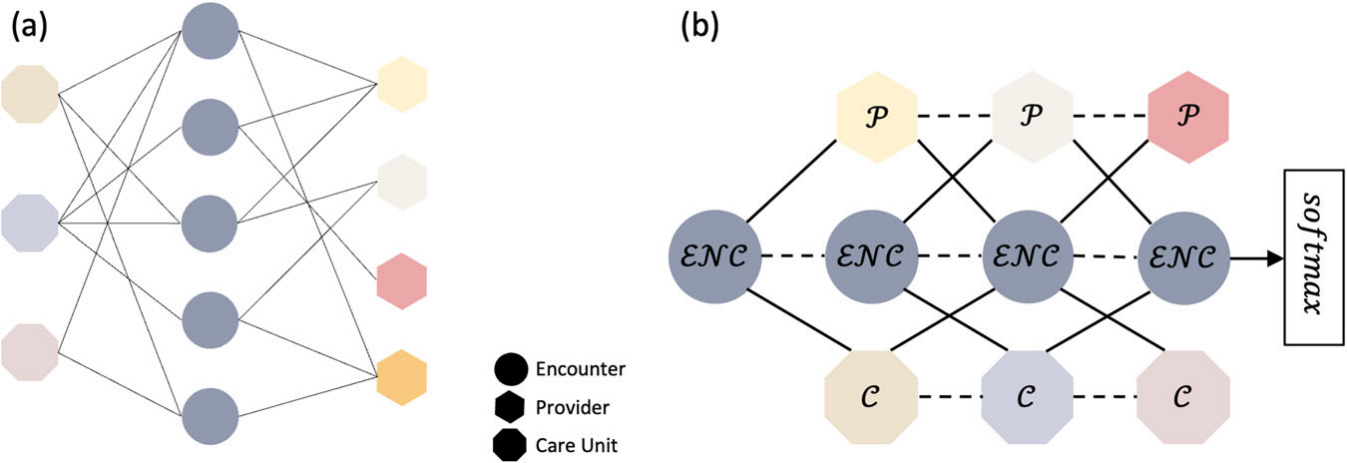
Graph representation and MedHG-PS model architecture. **a** The representation of the graph. Circles represent encounters, triangles represent providers, and squares represent care units. Different colors represent different providers or care unit types. **b** MedHG-PS with three stacked layers, where solid edges denote neighbor connections, and dashed edges represent self-relations.

A heterogeneous graph ${\mathcal{G}}={\mathcal{(}}{\mathcal{V}},{\mathcal{E}}{\mathcal{)}}$, as shown in Fig. 6a, was first constructed as the starting point using the input variables. The graph consists of three types of nodes ${\mathscr{V}}{\mathcal{\in }}{\mathcal{\{}}{\mathcal{E}}{\mathcal{N}}{\mathcal{C}},{\mathcal{P}},{\mathcal{C}}{\mathscr{\}}}$, representing encounter (${\mathcal{E}}{\mathcal{N}}{\mathcal{C}}$), provider (${\mathcal{P}}$), and care unit (${\mathcal{C}}$). All nodes are connected by undirected edges (${\mathcal{E}}$), which represent their interaction. For example, if a provider is associated with an encounter, a link is established between the corresponding nodes. Demographic and perioperative information were embedded into a feature matrix ${H}^{{\mathcal{E}}{\mathcal{N}}{\mathcal{C}}}\in {{\mathcal{R}}}^{{\mathcal{E}}{\mathcal{N}}{\mathcal{C}}{\mathcal{\times }}{\mathcal{F}}}$, where ${\mathcal{F}}$ is the number of features. The provider nodes (${\mathcal{P}}$) were divided into five subgroups: Surgical Team, Other Clinicians, Nurses, Technicians, and Others. The Surgical Team subgroup includes surgeons, surgical assistants, and surgical fellows/residents. Other Clinicians encompasses providers who do not directly perform surgery, such as anesthesiologists, certified registered nurse anesthetists, anesthesiologist assistants, residents, and physician assistants. The Nurse subgroup includes nurse monitors, nurse practitioners, post-anesthesia care unit nurses, pre-procedural nurses, registered nurses in interventional radiology, and scrub nurses. Technicians consist of cell saver technicians, control room technicians, cardiovascular technicians, gastrointestinal technicians, radiology technologists, surgical technologists, catheterization lab scrubs, and perfusionists. The care unit (${\mathcal{C}}$) was classified into three subgroups: Intermediate, Intensive, and Acute. ${\mathcal{P}}$ and ${\mathcal{C}}$ has no feature. For ablation studies, subgraphs only containing ${\mathcal{P}}$ and ${\mathcal{E}}{\mathcal{N}}{\mathcal{C}}$ without ${H}^{{\mathcal{E}}{\mathcal{N}}{\mathcal{C}}}$, ${\mathcal{P}}$ and ${\mathcal{E}}{\mathcal{N}}{\mathcal{C}}$ with ${H}^{{\mathcal{E}}{\mathcal{N}}{\mathcal{C}}}$, ${\mathscr{C}}$ and ${\mathcal{E}}{\mathcal{N}}{\mathcal{C}}$ with ${H}^{{\mathcal{E}}{\mathcal{N}}{\mathcal{C}}}$, was constructed.

An interpretable and efficient heterogeneous graph convolutional network (ie-HGCN)^16^ was implemented to model the heterogeneous graph ${\mathcal{G}}$. In the ie-HGCN, given the graph ${\mathcal{G}}$, the task is to learn representation matrices of nodes in ${\mathcal{E}}{\mathcal{N}}{\mathcal{C}}$ for health outcome prediction. The model architecture is illustrated in Fig. 6b, which contains three graph convolutional network (GCN) layers to learn embeddings layer-wise for each node $\Omega \in {\mathcal{E}}{\mathcal{N}}{\mathcal{C}}$, and then pass to softmax function to generate the outputs. In ie-HGCN, ${H}_{0}^{\Omega }$ refers to the input features, and each GCN layer calculated the node embeddings ${H}_{l}^{\Omega }$ based on previous-layer representation ${H}_{l-1}^{\Omega }$, where $l$ is the index of layer and $l\in \left[1,3\right]$, by aggregating self-features ${Z}^{\Omega }$ and neighboring features ${Z}^{\Gamma }$, where $\Gamma \in {{\mathcal{N}}}^{\Omega }=\{{n|n}\;{\mathscr{\in }}\,{\mathcal{V}}\thinspace{and}\thinspace{n}\;\ne \;\Omega \}$. Since the neighbors of a node in a heterogeneous graph are of different types, the convolution operation cannot be applied directly across all node types simultaneously. Instead, the features $Z$ was calculated separately by node types. The GCN layer can be formulated as Eq. (1):

1 $${H}_{l+1}^{\Omega }\,={ELU}\left({a}_{l}^{\Omega }\cdot {Z}_{l}^{\Omega }+\sum _{\Gamma \in {{\mathscr{N}}}^{\Omega }}{a}_{l}^{\Gamma }\cdot {Z}_{l}^{\Gamma }\right)$$

where ${ELU}(\cdot )$ is the Exponential Linear Unit activation function^47^. The normalized attention coefficients ${a}^{\Omega }$ and ${a}^{\Gamma }$ for each layer are calculated by Eq. (2). It follows the key, query, value attention mechanism maps a set of queries and a set of key-value pairs to an output. The attention function is implemented as follows:

2 $$\begin{array}{c}{a}^{\Omega }={{{softmax}}}({ELU}\,([({Z}^{\Omega }{W}_{k}^{\Omega }){{||}}({Z}^{\Omega }{W}_{q}^{\Omega })]\,\cdot {W}_{a}^{\Omega }))\\ {a}^{\Gamma }={{{softmax}}}({ELU}\,([({Z}^{\Gamma }{W}_{k}^{\Gamma }){{||}}({Z}^{\Omega }{W}_{q}^{\Omega })]\,\cdot {W}_{a}^{\Omega }))\end{array}$$

where trainable matrices ${W}_{k}$ maps $Z$ into attention keys, and ${W}_{q}$ maps $Z$ into attention queries. Operation || denotes the row-wise concatenation, and ${W}_{a}^{\Omega }$ is a learnable attention parameter vector. ${Z}^{\Omega }$ and ${Z}^{\Gamma }$ are generated by heterogeneous graph convolution operation. For ${Z}^{\Gamma }$, as is shown in Eq. (3), ${W}^{\Gamma -\Omega }$ along with the row‐normalized adjacency matrix ${\hat{A}}^{\Omega -\Gamma }={{{{diag}}}({A}^{\Omega -\Gamma })}^{-1}\cdot {A}^{\Omega -\Gamma }$ and the previous-layer representation ${H}_{l-1}^{\Gamma }$, together govern the transformation. For ${{Z}}^{\Omega }$, since the ${\hat{A}}^{\Omega -\Gamma }$ are identity matrix, the equation can be simplified to Eq. (4):

3 $${Z}^{\Gamma }={\hat{A}}^{\Omega -\Gamma }\cdot {H}_{l-1}^{\Gamma }\cdot {W}^{\Gamma -\Omega }$$

4 $${Z}^{\Omega }={H}_{l-1}^{\Omega }\cdot {W}^{\Omega -\Omega }$$

In the final step, the hidden embeddings from the third GNN layer are fed into a softmax function to generate the final prediction probabilities $\hat{y}$, as shown $\hat{y}={{\rm{softmax}}}\left({H}_{3}^{\Omega }\,\right)$. During the training phase, the objective is to minimize prediction errors. A gradient-based optimization method is employed to update the trainable parameters, which is illustrated below:

5 $${{{Loss}}}=\,-\frac{1}{N}\mathop{\sum }\limits_{i=1}^{N}\left[{y}_{{\rm{i}}}log \left({\hat{y}}_{{\rm{i}}}\right)+\left(1\,-\,{y}_{{\rm{i}}}\right)log \left(1\,-\,{\hat{y}}_{{\rm{i}}}\right)\right]$$

The significance of different input variables influencing patients’ safety outcomes were explored by MPA, SHAP^48^ analysis and LIME^49^. MPA assesses the impact of various paths in the graph structure across different node types on patient safety outcomes, offering insights into the most influential clinical interactions. To perform MPA, meta-paths from the graph was constructed by linking different node types. For example, a meta-path could be Encounter -> Provider -> Care unit. For each path $\Gamma -\Omega$, the mean normalized attention coefficients of edges ${a}_{l}^{\Gamma }$ from each model layer was recorded, capturing the interactions between specific node type pairs (e.g., Acute Care Unit and Encounter nodes). The overall importance of each meta-path was determined by multiplying the attention coefficients along the sequence of edges in the meta-path.

To identify the key demographic and perioperative information influencing patients’ safety outcomes, SHAP^48^ and LIME, widely used XAI techniques, were employed. SHAP and LIME allow for the interpretation of model predictions by quantifying the contribution of individual features. Given the high dimensionality of the feature space, the Clinical Classifications Software Refined (CCSR) database was applied to aggregate diagnosis and procedure codes into higher-level clinical categories, simplifying the features while preserving their clinical relevance^50^.

### Experimental settings

The proposed analysis was conducted on the University of Florida’s HiperGator high-performance computing cluster (https://www.rc.ufl.edu/about/hipergator/). The experiment used 16 cores of AMD EPYC 7742 processors, 64GB memory, and an Nvidia A100 GPU. For framework implementation and data processing, Python version 3.11 with the libraries: OpenHGNN^51^, XGBoost, Scikit-learn^52^, Scikit-optimize^53^, Captum^54^, MLstatkit, and Imbalanced-learn^55^ were used.

The MedHG-PS was compared with traditional ML models, including three representative ML algorithms: logistic regression (LR), multi-layer perceptron (MLP), and XGBoost^56^. These methods were evaluated using demographic and perioperative information alone, as well as in combination with different network science features to analyze provider interactions and patient transfers within the hospital.

To obtain network science features, the provider information of all hospital encounters was constructed as a single holistic provider-to-provider interaction graph, where nodes are individual providers and undirected edge, and the weights represent the number of interactions—two providers operated on the same patient. Then, the inpatient transfer information of each single hospital encounter was modeled as individual, standalone patient transfer graphs, where nodes represented hospital care units and directed edges indicated patient transfers between units (e.g., a patient from an acute care unit to an intensive care unit) during a single hospital stay. Various network metrics^57,58^ such as centralities, average degree connectivity, clustering coefficient, authority, page rank, density, number of nodes, and weighted number of edges were extracted from these graphs. For each encounter, these metrics were computed for each node (providers in the provider-to-provider interaction graph and care units in the patient transfer graphs) and then aggregated them into summary statistics, including maximum, minimum, mean, median, and interquartile range. As a result, the inputs for ML models included encounter-level demographics, perioperative information, and graph metrics derived from provider-to-provider interaction and patient transfer graphs.

Following ML best practices, the dataset was divided into training, validation, and testing sets with a ratio of 8:1:1 for model training and hyperparameter tuning. The detailed search space for hyperparameter tuning for different methods is summarized in Table 2. For the ie-HGCN in MedHG-PS, the Adam optimizer^59^ with binary cross-entropy loss (Eq. (4)) was used to train the MedHG-PS and tree-structured parzen estimator algorithm^60^ was used to optimize the learning rate, L2 regularization, dropout rate, batch normalization, the dimension of the first hidden layer, and attention vector. The training batch size was set to 512^61^.

**Table 2:** Hyperparameters

Model	Hyperparameter	Range
MedHG-PS	Learning rate	[10^−7^, 10^−1^]
	L2 regularization	[10^−7^, 10^−1^]
	Dropout rate	[0.0, 0.8]
	Batch normalization	{True, False}
	Dimension of the first hidden layer	{32, 64, 128, 256]
	Dimension of attention vector	{8, 16, 32, 64, 128}
XGBoost	Number of estimators	[50, 600]
	Learning rate	[0.01, 0.3]
	Max depth	[3, 15]
	Subsample	[0.6, 1]
	The fraction of features for training	[0.5, 1]
MLP	Learning rate scheduler	{constant, adaptive}
	L2 regularization	[0.0001, 0.99]
LR	C	[10^-−6^, 50]
	Class weight	{balance, none}

For the ML baselines, Bayes algorithm^62^ was used to tune hyperparameters and optimize performance. An early stopping strategy was applied to all training processes by monitoring the AUROC changes on the validation set. For the XGBoost, the L2 regularization was set to 1, and the L1 regularization was set to 0. For the MLP, the number of layers was set to 3, and the activation layers were ReLU^63^. The MLP was trained for a maximum of 200 iterations with the Adam optimizer with cross-entropy loss in an initial learning rate of 0.001. The training data was divided into mini-batches for MLP training, and the mini-batch size was 200. The LR models were optimized using the Newton-CG algorithm with L2 regularization with 500 iterations.

To evaluate the effectiveness of the imputation strategy, a masking approach was implemented based on previous studies. Prior research^64–66^ suggests that demographic information is generally well-recorded in EHR, with the exception of BMI, which may be missing at a rate of approximately 1.7% to 4.3%^67,68^, primarily due to the absence of height data. Marriage status data may also be missing at a rate of around 2.11%^64^. In addition, CCI might have a higher missing rate to 8.3%^69^. In this experiment, BMI and height were randomly masked at a rate of 4.3%, while marital status was masked at a rate of 2.11%. CCI was also masked at a rate of 8.3%. Given the advancements in EHR systems, most procedural intervals and calendar-related factors are recorded seamlessly. In addition, it remains challenging to distinguish between missing and truly negative for comorbidities, the presence of delirium, and the occurrence of anesthesia. Therefore, these features were not masked. Additionally, ASA-PS was masked at a rate of 1.7, consistent with previous findings^68^. The masked features were then imputed using the proposed methods.

All the models were evaluated on the testing set. Six typical metrics were adopted to assess model performance, including AUROC^70^, AUPRC^71^, F1-score, precision, recall^72^, and specificity^73^. To examinate the statistical significance between AUROC of different models, DeLong’s test^74^ was applied. 95% CI for AUROC and AUPRC were estimated using bootstrapping^75^.

## Supplementary information

Supplementary information

## Data Availability

Data from UF Health IDR can be requested through https://idr.ufhealth.org/research-services/data-request-form/. Since the UF Health data are a HIPAA-limited data set, a data use agreement needs to be established with the UF Health IDR research team.

## Code availability

The underlying code for this study is not publicly available but may be made available to qualified researchers on reasonable request from the corresponding author.

## References

[1] *Regional Strategy for Patient Safety in the WHO South-East Asia Region (2016–2025*) (World Health Organization, Regional Office for South–East Asia, 2015).

[2] James, J. T. A new, evidence-based estimate of patient harms associated with hospital care. *J. Patient Saf.* **9**, 122–128 (2013). 23860193 10.1097/PTS.0b013e3182948a69

[3] Liang, C. et al. Leveraging patient safety research: efforts made fifteen years since to err is human. *Stud. Health Technol. Inform.* **264**, 983–987 (2019). 31438071 10.3233/SHTI190371

[4] Kilic, A. et al. Predictive utility of a machine learning algorithm in estimating mortality risk in cardiac surgery. *Ann. Thorac. Surg.* **109**, 1811–1819 (2020). 31706872 10.1016/j.athoracsur.2019.09.049

[5] Tedesco, S. et al. Comparison of machine learning techniques for mortality prediction in a prospective cohort of older adults. *Int. J. Environ. Res. Public Health* **18**, 12806 (2021). 34886532 10.3390/ijerph182312806 PMC8657506

[6] Weiss, A. J. et al. Machine learning using institution-specific multi-modal electronic health records improves mortality risk prediction for cardiac surgery patients. *JTCVS Open* **14**, 214–251 (2023). 37425442 10.1016/j.xjon.2023.03.010 PMC10328834

[7] Vernooij, J. E. M. et al. Performance and usability of pre-operative prediction models for 30-day peri-operative mortality risk: a systematic review. *Anaesthesia* **78**, 607–619 (2023). 36823388 10.1111/anae.15988

[8] Rothen, H. U. & Takala, J. Can outcome prediction data change patient outcomes and organizational outcomes? *Curr. Opin. Crit. Care* **14**, 513–519 (2008). 18787442 10.1097/MCC.0b013e32830864e9

[9] Deimazar, G. & Sheikhtaheri, A. Machine learning models to detect and predict patient safety events using electronic health records: a systematic review. *Int. J. Med. Inform.* **180**, 105246 (2023). 37837710 10.1016/j.ijmedinf.2023.105246

[10] Brasel, K. J., Lim, H. J., Nirula, R. & Weigelt, J. A. Length of stay: an appropriate quality measure? *Arch. Surg.* **142**, 461–465 (2007). 17515488 10.1001/archsurg.142.5.461

[11] Baek, H. et al. Analysis of length of hospital stay using electronic health records: a statistical and data mining approach. *PLoS ONE* **13**, e0195901 (2018). 29652932 10.1371/journal.pone.0195901 PMC5898738

[12] Pronovost, P. J. et al. Framework for patient safety research and improvement. *Circulation* **119**, 330–337 (2009). 19153284 10.1161/CIRCULATIONAHA.107.729848

[13] Friend, T. H., Jennings, S. J., Copenhaver, M. S. & Levine, W. C. Implementation of the Vocera communication system in a quaternary perioperative environment. *J. Med. Syst.* **41**, 6 (2017). 27826766 10.1007/s10916-016-0652-9

[14] Gifford, E. & Foster, E. M. Provider-level effects on psychiatric inpatient length of stay for youth with mental health and substance abuse disorders. *Med. Care* **46**, 240–246 (2008). 18388838 10.1097/MLR.0b013e318158aee7

[15] Kim, C. et al. Provider networks in the neonatal intensive care unit associate with length of stay. *IEEE Conf. Collab. Internet Comput.* **2019**, 127–134 (2019). 32637942 10.1109/CIC48465.2019.00024 PMC7339831

[16] Yang, Y. et al. Interpretable and efficient heterogeneous graph convolutional network. *IEEE Trans. Knowl. Data Eng.* **35**, 1637–1650 (2021).

[17] Nasarian, E., Alizadehsani, R., Acharya, U. R. & Tsui, K.-L. Designing interpretable ML system to enhance trust in healthcare: a systematic review to proposed responsible clinician-AI-collaboration framework. *Inf. Fusion* **108**, 102412 (2024).

[18] Hicks, S. A. et al. On evaluation metrics for medical applications of artificial intelligence. *Sci. Rep.* **12**, 5979 (2022). 35395867 10.1038/s41598-022-09954-8 PMC8993826

[19] Li, R., Luo, Q., Green, D. & Huddleston, S. Smaller hospital size is associated with higher mortality in Stanford Type A Aortic Dissection. *Vasc. Endovascular Surg.* **59**, 5–11 (2025). 39185819 10.1177/15385744241278839

[20] Tipton, K. et al. Interventions To Decrease Hospital Length of Stay. *Technical Brief No. 40*. Rockville (MD): Agency for Healthcare Research and Quality. AHRQ Publication No. 21-EHC015. 10.23970/AHRQEPCTB40 (2021). 34644039

[21] Joung, R. H.-S. & Merkow, R. P. Is it time to abandon 30-day mortality as a quality measure? *Ann. Surg. Oncol.* **28**, 1263–1264 (2021). 33393040 10.1245/s10434-020-09262-3 PMC8148608

[22] Resio, B. J. et al. Where the other half dies: analysis of mortalities occurring more than 30 days after complex cancer surgery. *Ann. Surg. Oncol.* **28**, 1278–1286 (2021). 32885398 10.1245/s10434-020-09080-7

[23] Tarity, T. D. & Swall, M. M. Current trends in discharge disposition and post-discharge care after total joint arthroplasty. *Curr. Rev. Musculoskelet. Med.* **10**, 397–403 (2017). 28687957 10.1007/s12178-017-9422-7 PMC5577423

[24] Wasfy, J. H. et al. Predicting length of stay and the need for postacute care after acute myocardial infarction to improve healthcare efficiency. *Circ. Cardiovasc. Qual. Outcomes* **11**, e004635 (2018). 30354547 10.1161/CIRCOUTCOMES.118.004635 PMC6207219

[25] Gabriel, R. A. et al. A predictive model for determining patients not requiring prolonged hospital length of stay after elective primary total hip arthroplasty. *Anesth. Analg.* **129**, 43–50 (2019). 30234533 10.1213/ANE.0000000000003798

[26] Hirshon, J. M. et al. Health systems and services: the role of acute care. *Bull. World Health Organ.* **91**, 386–388 (2013). 23678202 10.2471/BLT.12.112664 PMC3646345

[27] Chen, L., Shi, L., Zhang, D., Jiang, C. & Truong, K. Does the “weekend effect” extend to Friday admissions? An analysis of ischemic stroke hospitalizations in South Carolina. *Front. Neurol.* **11**, 424 (2020). 32655467 10.3389/fneur.2020.00424 PMC7325933

[28] Major, P. et al. Risk factors for prolonged length of hospital stay and readmissions after laparoscopic sleeve gastrectomy and laparoscopic roux-en-Y gastric bypass. *Obes. Surg.* **28**, 323–332 (2018). 28762024 10.1007/s11695-017-2844-x PMC5778173

[29] Fletcher, R., Deal, R., Kubasiak, J., Torquati, A. & Omotosho, P. Predictors of increased length of hospital stay following laparoscopic sleeve gastrectomy from the national surgical quality improvement program. *J. Gastrointest. Surg.* **22**, 274–278 (2018). 29209980 10.1007/s11605-017-3642-4

[30] Smith, S. A. et al. Weekend surgical care and postoperative mortality: a systematic review and meta-analysis of cohort studies. *Med. Care* **56**, 121–129 (2018). 29251716 10.1097/MLR.0000000000000860 PMC5770102

[31] Greenburg, A. G., Saik, R. P. & Pridham, D. Influence of age on mortality of colon surgery. *Am. J. Surg.* **150**, 65–70 (1985). 4014573 10.1016/0002-9610(85)90011-x

[32] Sung, V. W., Weitzen, S., Sokol, E. R., Rardin, C. R. & Myers, D. L. Effect of patient age on increasing morbidity and mortality following urogynecologic surgery. *Am. J. Obstet. Gynecol.* **194**, 1411–1417 (2006). 16647926 10.1016/j.ajog.2006.01.050

[33] Veronese, N. & Maggi, S. Epidemiology and social costs of hip fracture. *Injury* **49**, 1458–1460 (2018). 29699731 10.1016/j.injury.2018.04.015

[34] Iacobucci, I. & Mullighan, C. G. Genetic basis of acute lymphoblastic leukemia. *J. Clin. Oncol.* **35**, 975–983 (2017). 28297628 10.1200/JCO.2016.70.7836 PMC5455679

[35] Charlson, M. E., Pompei, P., Ales, K. L. & MacKenzie, C. R. A new method of classifying prognostic comorbidity in longitudinal studies: development and validation. *J. Chronic Dis.* **40**, 373–383 (1987). 3558716 10.1016/0021-9681(87)90171-8

[36] Hennrikus, M., Hennrikus, W. P., Lehman, E., Skolka, M. & Hennrikus, E. The obesity paradox and orthopedic surgery. *Medicine* **100**, e26936 (2021). 34414951 10.1097/MD.0000000000026936 PMC8376337

[37] Gershman, B. et al. Comprehensive characterization of the perioperative morbidity of cytoreductive nephrectomy. *Eur. Urol.* **69**, 84–91 (2016). 26044802 10.1016/j.eururo.2015.05.022

[38] Tefera, G. M., Feyisa, B. B., Umeta, G. T. & Kebede, T. M. Predictors of prolonged length of hospital stay and in-hospital mortality among adult patients admitted at the surgical ward of Jimma University medical center, Ethiopia: prospective observational study. *J. Pharm. Policy Pract.* **13**, 24 (2020). 32549990 10.1186/s40545-020-00230-6 PMC7296702

[39] Liu, H. et al. Random forest predictive modeling of prolonged hospital length of stay in elderly hip fracture patients. *Front. Med.* **11**, 1362153 (2024). 10.3389/fmed.2024.1362153 PMC11140010 38828234

[40] Siracuse, J. J. et al. Risk factors for protracted postoperative length of stay after lower extremity bypass for critical limb ischemia. *Ann. Vasc. Surg.* **28**, 1432–1438 (2014). 24517986 10.1016/j.avsg.2013.12.027

[41] Gómez Hernández, M. T. et al. Predictive factors of prolonged postoperative length of stay after anatomic pulmonary resection. *Cir. Esp.* **101**, 43–50 (2023). 35787477 10.1016/j.cireng.2022.06.048

[42] Outcome and Payment Measures. https://www.cms.gov/medicare/quality/initiatives/hospital-quality-initiative/outcome-and-payment-measures (2024).

[43] Saklad, M. Grading of patients for surgical procedures. *Anesthesiology* **2**, 281–284 (1941).

[44] Zietlow, K. E. et al. Geriatric preoperative optimization: a review. *Am. J. Med.* **135**, 39–48 (2022). 34416164 10.1016/j.amjmed.2021.07.028 PMC8688225

[45] O’Leary, J. D. et al. Hospital admission on weekends for patients who have surgery and 30-day mortality in Ontario, Canada: a matched cohort study. *PLoS Med.* **16**, e1002731 (2019). 30695035 10.1371/journal.pmed.1002731 PMC6350956

[46] Deiwick, M. et al. Heart surgery in patients aged eighty years and above: determinants of morbidity and mortality. *Thorac. Cardiovasc. Surg.* **45**, 119–126 (1997). 9273957 10.1055/s-2007-1013702

[47] Clevert, D.-A., Unterthiner, T. & Hochreiter, S. Fast and accurate deep network learning by exponential linear units (ELUs). Preprint at http://arXiv.org/abs/1511.07289 (2015).

[48] Lundberg, S. & Lee, S.-I. A unified approach to interpreting model predictions. Preprint at https://arxiv.org/abs/1705.07874 (2017).

[49] Ribeiro, M. T., Singh, S. & Guestrin, C. Why should I trust you? in *Proceedings of the 22nd ACM SIGKDD International Conference on Knowledge Discovery and Data Mining* (ACM, 2016).

[50] *Clinical Classifications Software Refined (CCSR) for ICD-10-CM Diagnoses*. https://hcup-us.ahrq.gov/toolssoftware/ccsr/dxccsr.jsp. (2025).

[51] Han, H. et al. OpenHGNN: an open source toolkit for heterogeneous graph neural network. in *Proceedings of the 31st ACM International Conference on Information & Knowledge Management* (ACM, 2022).

[52] Pedregosa, F. et al. Scikit-learn: machine learning in Python. *J. Mach. Learn. Res.* **abs/1201.0490**, 2825–2830 (2011).

[53] Head, T., Kumar, M., Nahrstaedt, H., Louppe, G. & Shcherbatyi, I. Scikit-Optimize/Scikit-Optimize. *Zenodo*. 10.5281/ZENODO.5565057 (2021).

[54] Kokhlikyan, N. et al. Captum: a unified and generic model interpretability library for PyTorch. Preprint at https://arxiv.org/abs/2009.07896 (2020).

[55] Lemaître, G., Nogueira, F. & Aridas, C. K. Imbalanced-learn: a python toolbox to tackle the curse of imbalanced datasets in machine learning. *J. Mach. Learn. Res.* **abs/1609.06570**, 1–5 (2016).

[56] Chen, T. & Guestrin, C. XGBoost: a scalable tree boosting system. in *Proceedings of the 22nd ACM SIGKDD International Conference on Knowledge Discovery and Data Mining* (ACM, 2016).

[57] Wills, P. & Meyer, F. G. Metrics for graph comparison: a practitioner’s guide. *PLoS ONE* **15**, e0228728 (2020). 32050004 10.1371/journal.pone.0228728 PMC7015405

[58] Brunson, J. C. & Laubenbacher, R. C. Applications of network analysis to routinely collected health care data: a systematic review. *J. Am. Med. Inform. Assoc.* **25**, 210–221 (2018). 29025116 10.1093/jamia/ocx052 PMC6664849

[59] Kingma, D. P. & Ba, J. Adam: a method for stochastic optimization. Preprint at https://arxiv.org/abs/1412.6980 (2014).

[60] Watanabe, S. Tree-structured Parzen estimator: understanding its algorithm components and their roles for better empirical performance. Preprint at https://arxiv.org/abs/2304.11127 (2023).

[61] Hamilton, W. L., Ying, R. & Leskovec, J. Inductive representation learning on large graphs. Preprint at https://arxiv.org/abs/1706.02216 (2017).

[62] Bergstra, J., Bardenet, R., Bengio, Y. & Kégl, B. Algorithms for hyper-parameter optimization. *Neural Inf Process Syst* **24**, 2546–2554 (2011).

[63] Agarap, A. F. Deep learning using rectified linear units (ReLU). Preprint at https://arxiv.org/abs/1803.08375 (2018).

[64] Song, C., Luo, Z., Lu, L. & Su, Q. MLNet: enhancing joint predictive modeling of chronic diseases using deep learning. in *2023 IEEE International Conference on Bioinformatics and Biomedicine (BIBM)* 3165–3172 (IEEE, 2023).

[65] Bernardini, M., Doinychko, A., Romeo, L., Frontoni, E. & Amini, M.-R. A novel missing data imputation approach based on clinical conditional Generative Adversarial Networks applied to EHR datasets. *Comput. Biol. Med.* **163**, 107188 (2023). 37393785 10.1016/j.compbiomed.2023.107188

[66] Baer, H. J., Karson, A. S., Soukup, J. R., Williams, D. H. & Bates, D. W. Documentation and diagnosis of overweight and obesity in electronic health records of adult primary care patients. *JAMA Intern. Med.* **173**, 1648–1652 (2013). 23835808 10.1001/jamainternmed.2013.7815

[67] Lu, Y. et al. Leveraging the electronic health records for population health: a case study of patients with markedly elevated blood pressure. *J. Am. Heart Assoc.* **9**, e015033 (2020). 32200730 10.1161/JAHA.119.015033 PMC7428633

[68] Oh, T. K., Ryu, J.-H., Han, J.-W. O., Koo, C.-H. & Jeon, Y.-T. Factors associated with a 30-day unplanned readmission after elective spine surgery: a retrospective cohort study. *Eur. Spine J.* **30**, 191–199 (2021). 32754776 10.1007/s00586-020-06541-1

[69] Bhatia, A. et al. Effect of nirmatrelvir/ritonavir (Paxlovid) on hospitalization among adults with COVID-19: an EHR-based target trial emulation from N3C. Preprint at https://www.medrxiv.org/content/10.1101/2023.05.03.23289084v1 (2023). 10.1371/journal.pmed.1004493 PMC11790232 39823513

[70] Bradley, A. P. The use of the area under the ROC curve in the evaluation of machine learning algorithms. *Pattern Recognit.* **30**, 1145–1159 (1997).

[71] Boyd, K., Eng, K. H. & Page, C. D. Area under the Precision-Recall Curve: Point Estimates and Confidence Intervals. In *Machine Learning and Knowledge Discovery in Databases* (eds. Blockeel, H., Kersting, K., Nijssen, S. & Železný, F.) 451–466 (Springer Berlin Heidelberg, Berlin, Heidelberg, 2013).

[72] Lewis, D. D. Evaluating and optimizing autonomous text classification systems. in *Proceedings of the 18th Annual International ACM SIGIR Conference on Research and Development in Information Retrieval—SIGIR ’95* (ACM Press, 1995).

[73] Yerushalmy, J. Statistical problems in assessing methods of medical diagnosis, with special reference to X-ray techniques. *Public Health Rep.* **62**, 1432–1449 (1947). 20340527

[74] DeLong, E. R., DeLong, D. M. & Clarke-Pearson, D. L. Comparing the areas under two or more correlated receiver operating characteristic curves: a nonparametric approach. *Biometrics* **44**, 837–845 (1988). 3203132

[75] Efron, B. & Tibshirani, R. *An Introduction to the Bootstrap* (Springer, 1993).

