# Supplementary material for: Care coordination and patient safety outcome: a graph-based approach
Source: Npj Health Syst. 2025 May 8;2:15. doi: 10.1038/s44401-025-00020-9 (PMC13025955; doi:10.1038/s44401-025-00020-9)
Supplement: Supplementary file 1 — Supplementary information [file 44401_2025_20_MOESM1_ESM.pdf]

Supplemental materials

This supplementary file contains the additional section and tables of the experimental results for readers. The content is outlined as follows:

Supplementary Section 1: Related Work

Supplementary Table S1. Comparison with previous studies

Supplementary Table S2. Statistics of the Study Cohort

Supplementary Table S3. Missing rate of non-binary features

Supplementary Table S4. Model Performance in Predicting Prolonged Length of Stay

Supplementary Table S5. Model Performance in Predicting 30-day Mortality

Supplementary Table S6. Model Performance in Predicting 90-day Mortality

Reference

Related Works

As part of the learning health system, research targeting improving patient outcomes and quality of care through optimizing clinical care processes is urgently needed[1]. However, this is a complex task due to the multitude of factors influencing patient outcomes, including not only demographic variables and clinical factors[2] but also care coordinations[3]. As is shown in **Supplementary Table S1**, Current strategies for predicting patient safety outcomes can be categorized into two primary approaches: patient features-based and coordination features-based methodologies.

Patient features-based approaches primarily focus on predicting safety outcomes based on individual patient characteristics. The pioneering work by Sund et al.[4] developed a Bayesian nonparametric multilayer perceptron network model that utilized demographic variables and clinical factors, such as age and hip fracture type, to predict length of stay. Soon after, Lin et al.[5] developed logistic regression and artificial neural network models to predict one-year mortality. DeBaun et al.[6] expanded the scope by incorporating additional pre-operative features into machine learning models to predict 30-day mortality. Murase et al.[7] developed sophisticated deep learning models to predict prolonged length of stay (PLOS) using a comprehensive set of variables including demographics, comorbidities, medications, laboratory data, physical examinations, and surgical procedure details. Most recently, Chung et al.[8] evaluated the capabilities of large language model in predicting multiple patient safety outcome, including American Society of Anesthesiologists physical status classification system (ASA-PS), hospital admission, ICU admission, unplanned admission, and hospital mortality, based on clinical notes. Despite these advancements, a significant limitation persists: previous studies have predominantly focused on single surgical sites and utilized only clinical features, neglecting the critical dimension of provider coordination.

Coordination features-based strategies focus on analyzing the influence of provider coordination on patient safety outcomes. Wrobel et al. developed the Foot Systems Assessment Tool (FootSAT) to measure provider coordination across four key dimensions: programming coordination, feedback coordination, resources and information support. [9] Their results demonstrated a strong association between FootSAT scores and amputation rates. Similarly, Hallet et al.[10] investigated the relationship between the familiarity of the surgeon-anesthesiologist team and postoperative outcomes in complex gastrointestinal cancer surgeries. Their findings indicated that each additional surgery in which the same surgeon and anesthesiologist worked together reduced the odds of 90-day major morbidity by 5%. However, these studies typically utilized a limited number of features to describe coordination quality for statistical analysis. Notably, graph science methodologies—powerful tools for modeling complex relationships—have not previously been applied to model care coordination for patient safety outcomes.

To explore the impact of multiple factors (e.g., demographic, clinical, and coordination factors) on predicting diverse surgical outcomes, the MedHG-PS model was proposed, leveraging graph modeling techniques. Notably, the inclusion of heterogeneous graph modeling to reflect provider-provider interactions and patient transfers between care units significantly improved outcome predictions.

Supplementary Table S1. Comparison with previous studies

Study	Patient Features		Coordination Features	Model	Targeted Population	Target	
	Demographics	Clinical Factors				Prolonged Length of Stay	Mortality
Sund et al.	✓	✓	✗	Bayesian nonparametric multilayer perceptron network model	Hip fracture	✓	✗
Lin et al.	✓	✓	✗	Logistic regression; artificial neural network model	Hip fracture	✗	✓
DeBaun et al.	✓	✓	✗	Artificial neural network; naive Bayes; logistic regression	Hip fracture	✗	✓
Murase et al.	✓	✓	✗	Deep learning model	Cardiovascular surgery	✓	✗
Chung et al.	✗	✓	✗	Large language model	All surgery	✓	✓
Wrobel et al.	✗	✗	✓	-	Diabetes	✗	✗
Hallet et al.	✗	✗	✓	-	Gastrointestinal cancer surgery	✗	✓
This study	✓	✓	✓	Graph convolutional network	All surgery	✓	✓

Supplementary Table S2. Statistics of the Study Cohort

	Total	PLOS	Non-PLOS	*p*-value	30D-Death	Non-30D-Death	*p*-value	90D-Death	Non-90D-Death	*p*-value
**Age**				<0.01			<0.01			<0.01
**[0,18)**	0(0.00%)	0(0.00%)	0(0.00%)		0(0.00%)	0(0.00%)		0(0.00%)	0(0.00%)	
**[18,50)**	44,531(32.59%)	8,809(25.79%)	35,722(34.85%)		683(15.81%)	43,848(33.14%)		1,059(14.56%)	43,472(33.60%)	
**50+**	92,116(67.41%)	25,347(74.21%)	66,769(65.15%)		3,636(84.19%)	88,480(66.86%)		6,215(85.44%)	85,901(66.40%)	
**Sex**				<0.01			<0.01			<0.01
**Female**	69,416(50.80%)	15,063(44.10%)	54,353(53.03%)		1,789(41.42%)	67,627(51.11%)		3,093(42.52%)	66,323(51.26%)	
**Male**	67,231(49.20%)	19,093(55.90%)	48,138(46.97%)		2,530(58.58%)	64,701(48.89%)		4,181(57.48%)	63,050(48.74%)	
**Race-Ethnicity**				<0.01			<0.01			<0.01
**NHW**	101,455(74.25%)	25,294(74.05%)	76,161(74.31%)		3,247(75.18%)	98,208(74.22%)		5,501(75.63%)	95,954(74.17%)	
**NHB**	21,130(15.46%)	5,662(16.58%)	15,468(15.09%)		586(13.57%)	20,544(15.53%)		1,016(13.97%)	20,114(15.55%)	
**Hispanic**	6,988(5.11%)	1,540(4.51%)	5,448(5.32%)		153(3.54%)	6,835(5.17%)		242(3.33%)	6,746(5.21%)	
**Other**	7,074(5.18%)	1,660(4.86%)	5,414(5.28%)		333(7.71%)	6,741(5.09%)		515(7.08%)	6,559(5.07%)	
**Marital Status**				<0.01			<0.01			<0.01
**Single**	42,246(30.92%)	10,509(30.77%)	31,737(30.97%)		1,114(25.79%)	41,132(31.08%)		1,824(25.08%)	40,422(31.24%)	
**Married**	62,111(45.45%)	14,662(42.93%)	47,449(46.30%)		1,932(44.73%)	60,179(45.48%)		3,309(45.49%)	58,802(45.45%)	
**Life Partner/Significant Other**	1,331(0.97%)	294(0.86%)	1,037(1.01%)		27(0.63%)	1,304(0.99%)		50(0.69%)	1281-0.0099	
**Separated**	1,421(1.04%)	388(1.14%)	1,033(1.01%)		35(0.81%)	1,386(1.05%)		71(0.98%)	1,350(1.04%)	
**Divorced**	11,314(8.28%)	3,239(9.48%)	8,075(7.88%)		416(9.63%)	10,898(8.24%)		712(9.79%)	10,602(8.19%)	
**Widowed**	8,444(6.18%)	2,529(7.40%)	5,915(5.77%)		425(9.84%)	8,019(6.06%)		779(10.71%)	7,665(5.92%)	
**Other**	9,780(7.16%)	2,535(7.42%)	7,245(7.07%)		370(8.57%)	9,410(7.11%)		529(7.27%)	9,251(7.15%)	
**Total**	136,647	34,156	102,491		4,319	132,328		7,274	129,373	

Supplementary Table S3. Missing rate of non-binary features

Feature	Missing Rate
Day of Week	0.00%
Race	0.01%
Ethnicity	0.01%
Sex	0.00%
Marital Status	1.23%
Service	0.47%
Anesthesia Type	0.00%
Admit Source	0.20%
Payer	0.00%
Age	0.00%
ASA PS	1.01%
CCI	28.49%
Weight	7.29%
Height	9.22%
BMI	9.52%

Supplementary Table S4. Model Performance in Predicting Prolonged Length of Stay

		AUROC	F1	Precision	Recall	Specificity
Demographics + Perioperative	XGBoost	**0.964**	0.827	**0.843**	0.812	**0.938**
	MLP	0.958	**0.833**	0.808	**0.860**	0.917
	LR	0.895	0.732	0.687	0.784	0.855
Provider Team	XGBoost	0.645	0.272	0.581	0.178	0.948
	MLP	0.634	0.180	**0.660**	0.104	**0.978**
	LR	0.623	0.448	0.289	**1.000**	0.000
	MedHGN-PS	**0.694**	**0.503**	0.415	0.640	0.634
Demographics + Perioperative + Provider Team	XGBoost	0.962	0.823	**0.853**	0.795	**0.945**
	MLP	0.942	0.688	0.842	0.581	0.956
	LR	0.903	0.731	0.640	0.852	0.806
	MedHGN-PS	**0.965**	**0.846**	0.772	**0.936**	0.888
Demographics + Perioperative +Patient Transfer	XGBoost	**0.972**	0.849	**0.860**	0.837	**0.945**
	MLP	0.967	**0.853**	0.829	0.880	0.926
	LR	0.900	0.739	0.688	0.798	0.853
	MedHGN-PS	0.968	0.851	0.786	**0.927**	0.898
Demographics + Perioperative + Provider Team + Patient Transfer	XGBoost	**0.971**	**0.845**	**0.872**	0.819	0.951
	MLP	0.944	0.628	0.868	0.492	**0.970**
	LR	0.907	0.743	0.671	0.833	0.834
	MedHGN-PS	0.960	**0.844**	0.786	**0.912**	0.900
Undersampled Demographics + Perioperative + Provider Team + Patient Transfer	XGBoost	**0.969**	**0.851**	0.787	**0.927**	0.898
	MLP	0.948	0.672	**0.887**	0.541	**0.972**
	LR	0.903	0.739	0.676	0.815	0.841
	MedHGN-PS	0.957	0.841	0.786	0.904	0.900
Oversampled Demographics + Perioperative + Provider Team + Patient Transfer	XGBoost	**0.971**	**0.855**	**0.785**	**0.938**	0.896
	MLP	0.947	0.816	0.752	0.890	0.881
	LR	0.908	0.744	0.671	0.836	0.833
	MedHGN-PS	0.945	0.810	0.772	0.851	**0.898**
Masked Demographics + Perioperative + Provider Team + Patient Transfer	XGBoost	**0.971**	**0.843**	**0.877**	0.811	0.954
	MLP	0.941	0.698	0.857	0.588	**0.960**
	LR	0.907	0.742	0.670	0.832	0.833
	MedHGN-PS	0.951	0.842	0.768	**0.932**	0.886

Supplementary Table S5. Model Performance in Predicting 30-day Mortality

		AUROC	F1	Precision	Recall	Specificity
Demographics + Perioperative	XGBoost	**0.916**	**0.332**	**0.821**	0.208	**0.998**
	MLP	0.898	0.294	0.772	0.181	**0.998**
	LR	0.784	0.208	0.126	**0.592**	0.849
Provider Team	XGBoost	0.649	0.000	0.000	0.000	**1.000**
	MLP	0.601	0.000	0.000	0.000	**1.000**
	LR	0.591	0.073	0.038	**0.847**	0.209
	MedHGN-PS	**0.721**	**0.124**	0.068	0.682	**0.658**
Demographics + Perioperative + Provider Team	XGBoost	**0.914**	0.309	**0.867**	0.188	0.999
	MLP	0.808	0.012	0.600	0.006	**1.000**
	LR	0.809	0.187	0.109	0.664	0.800
	MedHGN-PS	0.905	0.285	0.175	**0.777**	0.865
Demographics + Perioperative +Patient Transfer	XGBoost	**0.917**	**0.325**	0.798	0.204	0.998
	MLP	0.908	0.226	**0.875**	0.130	**0.999**
	LR	0.788	0.207	0.126	0.596	0.847
	MedHGN-PS	0.904	0.261	0.155	**0.825**	0.835
Demographics + Perioperative + Provider Team + Patient Transfer	XGBoost	**0.914**	0.315	**0.877**	0.192	0.999
	MLP	0.855	0.008	0.667	0.004	**1.000**
	LR	0.809	0.188	0.109	0.670	0.798
	MedHGN-PS	0.901	0.314	0.197	**0.771**	0.884
Undersampled Demographics + Perioperative + Provider Team + Patient Transfer	XGBoost	**0.911**	**0.438**	**0.417**	0.462	**0.976**
	MLP	0.845	0.327	0.251	0.468	0.949
	LR	0.806	0.177	0.102	0.662	0.785
	MedHGN-PS	0.805	0.157	0.088	**0.755**	0.711
Oversampled Demographics + Perioperative + Provider Team + Patient Transfer	XGBoost	**0.903**	**0.407**	0.328	0.536	0.960
	MLP	0.869	0.334	0.252	0.497	0.946
	LR	0.814	0.188	0.109	**0.680**	0.795
	MedHGN-PS	0.884	0.361	**0.530**	0.274	**0.991**
Masked Demographics + Perioperative + Provider Team + Patient Transfer	XGBoost	**0.913**	**0.301**	**0.840**	0.184	0.999
	MLP	0.845	0.012	0.333	0.006	**1.000**
	LR	0.805	0.187	0.109	0.662	0.801
	MedHGN-PS	0.903	0.289	0.176	**0.800**	0.862

Supplementary Table S6. Model Performance in Predicting 90-day Mortality

		AUROC	F1	Precision	Recall	Specificity
Demographics + Perioperative	XGBoost	**0.898**	**0.297**	0.673	**0.191**	0.995
	MLP	0.882	0.219	**0.686**	0.131	**0.997**
	LR	0.812	0.282	0.179	0.674	0.824
Provider Team	XGBoost	0.603	0.000	0.000	0.000	**1.000**
	MLP	0.517	0.000	0.000	0.000	**1.000**
	LR	0.559	0.109	0.059	0.672	0.394
	MedHGN-PS	**0.701**	**0.155**	**0.087**	**0.718**	**0.570**
Demographics + Perioperative + Provider Team	XGBoost	**0.896**	**0.311**	**0.665**	0.203	0.994
	MLP	0.832	0.011	0.444	0.005	**1.000**
	LR	0.824	0.267	0.167	**0.663**	0.812
	MedHGN-PS	0.701	0.174	0.102	0.601	0.698
Demographics + Perioperative +Patient Transfer	XGBoost	**0.899**	**0.308**	**0.724**	0.196	**0.996**
	MLP	0.883	0.229	0.687	0.137	**0.996**
	LR	0.814	0.281	0.177	0.672	0.823
	MedHGN-PS	0.898	0.302	0.184	**0.834**	0.790
Demographics + Perioperative + Provider Team + Patient Transfer	XGBoost	**0.895**	**0.306**	0.678	0.197	0.995
	MLP	0.820	0.000	0.000	0.000	1.000
	LR	0.830	0.267	0.166	0.690	0.803
	MedHGN-PS	**0.895**	0.303	0.185	**0.827**	0.793
Undersampled Demographics + Perioperative + Provider Team + Patient Transfer	XGBoost	**0.892**	**0.252**	**0.147**	**0.871**	0.713
	MLP	0.836	0.243	0.143	0.793	**0.731**
	LR	0.819	0.218	0.126	0.814	0.680
	MedHGN-PS	0.753	0.196	0.116	0.627	0.728
Oversampled Demographics + Perioperative + Provider Team + Patient Transfer	XGBoost	**0.880**	**0.335**	0.217	0.732	0.850
	MLP	0.859	0.292	0.181	**0.754**	0.807
	LR	0.834	0.267	0.164	0.706	0.796
	MedHGN-PS	0.857	0.011	**0.286**	0.005	**0.999**
Masked Demographics + Perioperative + Provider Team + Patient Transfer	XGBoost	**0.896**	**0.315**	**0.703**	0.203	0.995
	MLP	0.815	0.003	0.500	0.001	**1.000**
	LR	0.829	0.267	0.165	**0.690**	0.802
	MedHGN-PS	0.787	0.220	0.132	0.653	0.756

Reference

1. Wouters, R. H. P., van der Graaf, R., Voest, E. E. & Bredenoord, A. L. Learning health care systems: Highly needed but challenging. *Learn. Health Syst.* **4**, e10211 (2020).
2. Deimazar, G. & Sheikhtaheri, A. Machine learning models to detect and predict patient safety events using electronic health records: A systematic review. *Int. J. Med. Inform.* **180**, 105246 (2023).
3. Joseph, S., Tomaschek, R., Hug, B. L. & Beeler, P. E. Enhancing communication and care coordination: A scoping review of encounter notification systems between emergency departments and primary care providers. *Int. J. Med. Inform.* **191**, 105579 (2024).
4. Klemt, C. *et al.* Artificial intelligence algorithms accurately predict prolonged length of stay following revision total knee arthroplasty. *Knee Surg. Sports Traumatol. Arthrosc.* **30**, 2556–2564 (2022).
5. Lin, C.-C., Ou, Y.-K., Chen, S.-H., Liu, Y.-C. & Lin, J. Comparison of artificial neural network and logistic regression models for predicting mortality in elderly patients with hip fracture. *Injury* **41**, 869–873 (2010).
6. DeBaun, M. R. *et al.* Artificial neural networks predict 30-day mortality after hip fracture: Insights from machine learning. *J. Am. Acad. Orthop. Surg.* **29**, 977–983 (2021).
7. Murase, R., Shingu, Y. & Wakasa, S. A preliminary prediction model using a deep learning software program for prolonged hospitalization after cardiovascular surgery. *Surg. Today* **53**, 393–395 (2023).
8. Chung, P. *et al.* Large language model capabilities in perioperative risk prediction and prognostication. *JAMA surgery* vol. 159 928–937 (2024).
9. Wrobel, J. S. *et al.* The relationship between provider coordination and diabetes-related foot outcomes. *Diabetes Care* **26**, 3042–3047 (2003).
10. Hallet, J. *et al.* Association between familiarity of the surgeon-anesthesiologist dyad and postoperative patient outcomes for complex gastrointestinal cancer surgery. *JAMA Surg.* **158**, 465–473 (2023).
